# Effector Memory T Cells and CD45RO+ Regulatory T Cells in Metastatic vs. Non-Metastatic Lymph Nodes in Lung Cancer Patients

**DOI:** 10.3389/fimmu.2022.864497

**Published:** 2022-05-02

**Authors:** Iwona Kwiecień, Elżbieta Rutkowska, Rafał Sokołowski, Joanna Bednarek, Agata Raniszewska, Karina Jahnz-Różyk, Piotr Rzepecki, Joanna Domagała-Kulawik

**Affiliations:** ^1^ Department of Internal Medicine and Hematology, Laboratory of Flow Cytometry, Military Institute of Medicine, Warsaw, Poland; ^2^ Department of Internal Medicine, Pulmonology, Allergology and Clinical Immunology, Military Institute of Medicine, Warsaw, Poland; ^3^ Department of Internal Medicine and Hematology, Military Institute of Medicine, Warsaw, Poland; ^4^ Department of Internal Medicine, Pulmonary Diseases and Allergy, Medical University of Warsaw, Warsaw, Poland

**Keywords:** lymph nodes, CD4+ cells, CD8+ cells, EBUS-TBNA, regulatory T cells, recent thymic emigrants cells, effector memory T cells, Th17- related cells

## Abstract

Lymphocytes play a leading role in regulation of the immune system in lung cancer patients. The recognition of T cells profile may help in prediction of effectiveness of anticancer immunotherapy. The aim of the study was to determine the dominant subpopulation of CD4+ and CD8+ lymphocytes in metastatic and non-metastatic lymph nodes (LNs) of lung cancer patients. LNs aspirates were obtained during EBUS/TBNA procedure and cells were analyzed by flow cytometry. We showed a higher percentage of CD4+ and CD8+ effector memory T cells in the metastatic than in the non-metastatic LNs (28.6 vs. 15.3% and 28.6 vs. 14.0%, p< 0.05). The proportion of CD45RO+ T regulatory cells (CD45RO+ Tregs) was higher in the metastatic LNs than in the non-metastatic ones (65.6 vs. 31%, p< 0.05). We reported the significant differences in T cell subsets depending on the lung cancer metastatic process. We observed that the effector memory T cells were predominant subpopulations in metastatic LNs. Lymphocyte profile in LNs is easy to evaluate by flow cytometry of EBUS/TBNA samples and may reflect the immune status in lung cancer.

## Introduction

### Lung Cancer

Lung cancer is the most common cause of cancer death in the world, with 2,200,000 new cases a year (1). Taking into account the different clinical course, cell biology, and treatment method, two main histological types of lung cancer are distinguished: non-small cell lung cancer (NSCLC) and small cell lung cancer (SCLC). According to the classification, the NSCLC group includes squamous cell carcinoma (SQCLC), adenocarcinoma (ADC) and the NOS subtype (not otherwise specified, recognized in a small fraction of samples) (2). As a solid tumor with low antigen specificity, lung cancer escapes immune surveillance and attacks by cytotoxic lymphocytes cells which play a major role in the anti-neoplastic defense of the immune system (3).

The presence of neoplastic metastases in the mediastinal lymph nodes (LNs) is one of the most important elements in determining the optimal treatment strategy in patients with NSCLC (4). In recent years, the mediastinal LNs are increasingly researched by use of ultrasound-guided transbronchial needle aspiration (EBUS/TBNA) technique with, routinely, evaluation in light microscopy, but it can provide high diagnostic performance with using flow cytometry analysis (5, 6). It is known that tests performed with the use of peripheral blood (PB) do not show local changes in lung cancer microenvironment. PB serves as a material for the assessment of systemic changes while EBUS/TBNA node biopsy is the material that allows to obtain rich cellular material from the nearest disease area reflecting the tumor microenvironment (TME) (7).

### LNs and Lymphocyte Phenotype

LNs act as an initiation site for the immune response and primary site for antigen presentation and lymphocyte maturation (8). Migrating naive thymic T cells called recent thymic emigrants (RTEs) mature into naive T lymphocytes after reaching the node and upon contact with the antigen presented by dendritic cells (DCs).

T naïve cells CD3+ CD45RA+ CD45RO- CD197+ differentiate into short−term living effector T cells phenotypically characterized as CD3+ CD45RA+ CD45RO- CD197-. They are heterogeneous population cells which produce cytokines and cytotoxic molecules, show activation markers and are involved in the inflammatory response (9). These cells have the potential to differentiate into long-lived memory cells which subsets were originally defined based on homing and migration markers along with effector functions. Central memory cells are phenotypically characterized as CD3+ CD45RA- CD45RO+ CD197+ (10, 11), express the LNs homing receptors CCR7 and CD62L, possess a high proliferative capacity but low cytotoxicity and after restimulation are activated, proliferate, and form immune memory. They differentiate into T effector cells (reaching inflammatory tissues) and a smaller population of central memory T cells (circulating between lymphatic organs) (12, 13). Memory T effector cells with the phenotype CD3+ CD45RA- CD45RO+ CD197- with inflammatory capacity, migration potential, and effector function generally do not migrate to LNs and only a very small fraction has been recently found in the LNs (9). Recent reports indicate a large variety of memory cells, including: terminally differentiated effector memory (Temra) cells, memory stem (Tscm) cells, and tissue-resident memory (Trm) cells (14, 15). Temra cells are a heterogeneous population, and although they are memory effector cells, they acquire expression of the CD45RA antigen upon antigenic stimulation and are involved in the immune response (16).

It has been found that infiltration of metastatic lymph nodes by memory T cells is an independent positive prognostic factor in patients with NSCLC (17). It is known that tumor infiltrating naive T cells differentiate into functional effector cells in the tumor microenvironment and promote tumor cells destruction (18, 19). CD8+ T cells with effector markers have been identified in cancer patients and appear to overlap with depleted T cells at tumor sites (20, 21).

The human CD4+ T cell is a heterogeneous population, divided into sub-populations depending on the functions performed. The activities of individual subpopulations result from the type of transcription factors and receptors contained in cells, as well as secreted cytokines, specific for each subpopulation (22).

In addition to the well-known Th1 and Th2 subpopulations, as well as Treg regulatory lymphocytes, additional subpopulations have been described. Th9, Th17, and Th22 are marked according to the type of secreted “main” cytokine and T follicular helper occurring in lymphoid organs (23).

### T Cells of Regulatory Properties

Studies conducted in recent years have shown that other subpopulations of CD4+ T lymphocytes also participate in the polarization of the specific response to tumor cells: T regulatory cells (Tregs) and Th17 cells. Tregs play the most important role in regulating the immune response in tumors to inhibit the activity of T cells (CD4+, CD8+), dendritic cells, and NK cells (24). Tregs play a key role in tumor immunology, having an important impact on the outcome of cancer patients (25). The negative prognostic significance of the presence of Tregs has been confirmed in numerous studies on lung cancer (26). Studies reported significantly higher percentage of Tregs in patients with metastatic NSCLC compared to healthy donors with correlation to more advanced stage of disease (27–29). It has been proposed to differentiate Tregs to naive, effector, and terminal effector subtype on the basis of the expression of CD45RO marker (30). However, the prognosis impact of different Tregs subsets in lung cancer prognosis it is limited, sometimes controversial, and additional studies on the Tregs subsets appear to be interesting. In addition, it is also interesting to evaluate Th17 cells, which may play a role in the anti-tumor response. These cells belong to the group of CCR6 + (CD196 +) memory T-helper cells (CCR6 + memTh). CCR6 + memTh is a heterogeneous population that contains Th17/Th22 and Th17/Th1 cells (31). These cells produce pro-inflammatory cytokines such as interleukin (IL)-17A, tumor necrosis factor alpha (TNFα), and interferon-gamma (IFNγ) (32–34). From the above population of T lymphocytes, Th17 may remain an important prognostic factor due to some reports on the role of these cells in the neoplastic environment. Th17 lymphocytes play an important role in antimicrobial and antifungal defense but have weak proliferative and cytotoxic properties (35). Their role in autoimmune processes has been proven, but there are no unambiguous data on the anti-tumor activity of Th17 lymphocytes, although studies show the presence of these cells in the tumor microenvironment (36). It seems that the function of Th17 cells and interleukin-17 (IL-17) produced by Th17 cells, due to its pleiotropism, may have an inhibitory or stimulating effect. The stimulatory effect may be related to the pro-angiogenic effect of IL-17 (37). The antitumor activity of these cells has been demonstrated in numerous studies which showed that IL-17 induces a specific antitumor response and leads to tumor growth inhibition (38). The cells with the phenotype are CD3+ CD4+ CD45RO+ CD196+ which we assessed belong to the memTh CCR6 + cell group. Due to the lack of functional tests to assess cytokines or assay of cell cultures, we called them Th17-related cells.

### Aim of the Study

The cellular characteristics of the lymph node and the definition of T-lymphocyte subpopulation in the affected lymph node and in the metastatic-free node seem interesting. Based on previous studies, it is known that the lung environment shows a certain dissimilarity in terms of the nature of the immune response (26, 39–41).

The aim of this study was to determine the dominant subtypes of CD4+ and CD8+ lymphocytes in metastatic and non-metastatic LNs of lung cancer patients. Accurate cellular assessment of lymph nodes offers the possibility of setting a new direction in the pre-therapeutic evaluation of lung cancer patients and the development of additional prognostic markers. Understanding cellular elements that play a role in the immediate disease environment that enables EBUS aspirate testing may have important therapeutic implications.

## Materials and Methods

### Patients

Patients with histologically confirmed primary lung cancer were included in the study groups (n = 30) (the current histological classification (2) and the 8th edition of TNM classification of lung cancer were used (42)). The study group consisted of patients undergoing diagnostic procedures for lung tumors. Patients without any type of previous or recent anticancer therapy, clinical signs of infection, autoimmune diseases, or immunosuppressive treatment were qualified for the study. 

Each patient had provided written informed consent (the Military Institute of Medicine Ethics Committee: 25/WIM/2018) before the diagnostic procedure which included EBUS/TBNA and PB collection. The individual clinical characteristic of the lung cancer patients is summarized in **Table 2**.

Before EBUS/TBNA, chest computed tomography was performed. During EBUS, suspected metastatic LNs were punctured beginning from the most distal node station. When the quality of the sample was appropriate, the material was divided for cytopathology staining, molecular testing, and flow cytometry analysis.

LNs were considered metastatic when the standard cytopathology samples were classified as positive, histology was consistent with primary tumor, or results of EBUS/TBNA provided a clear diagnosis of cancer.

After precise determination of histological type of lung cancer, the evaluation of biomarkers for further therapy was done according to recommendations (43, 44). Molecular analysis was performed in 4 patients with adenocarcinoma, 1 with not otherwise specified NOS type of lung cancer, 1 adenosquamous, and 2 with squamous type. PD-L1 expression was performed in 9 patients. The decision of evaluation of molecular tests or PD-L1 expression depended on therapeutic plan and patient performance status. Thus, the number of patients with biomarker analysis was low (presented for individual patients in **Table 2**).

The consecutive samples were collected for flow cytometry analysis and only those that fulfilled inclusion criteria were chosen for further analysis. Next, we divided the samples according to the presence of LNs metastases to metastatic LNs and those without cancer cells (non-metastatic).

### Materials

The aspirates from the LNs group 4, 7, 10, and 11 were obtained during the routine EBUS/TBNA procedure of lung cancer diagnosis. After diagnostic aspiration, an additional sample was taken for a flow cytometry analysis. About 1 ml of LNs aspirates was diluted in 0.9% NaCl, collected in tubes containing K_2_EDTA, and processed for flow cytometry. 2 ml of peripheral blood (PB) was taken and placed it in tubes containing K_2_EDTA and processed for flow cytometry.

### Flow Cytometry Analysis

For flow cytometric analysis 100 μL of LNs aspirate, the same volume of PB and 4 μL of specific monoclonal antibodies were added to each cytometric tube for surface marker detection. Cells were stained with fluorescently labeled antibodies for 20 min at room temperature. Erythrocytes were lysed with Pharm Lyse Lysing Buffer (BD Biosciences, Franklin Lakes, NJ, USA) for 10 min. After washing, cells were analyzed within 2 h. For each sample, a minimum of 100,000 events were collected using the FACS Canto II BD flow cytometry (BD Biosciences). The data were analyzed with DIVA Analysis software 8.0.1 (BD Biosciences) and Infinicyt 1.8 Flow Cytometry (Cytognos, Salamanca, Spain).

To evaluate the T lymphocyte subpopulation and main leukocytes, subsets of the following antibodies were used:

CD45-V500-C (catalog number 655873, clone number: 2D1, BD Biosciences), CD3-PerCP-Cy5.5 (catalog number: 332771, clone number: SK7, BD Biosciences), CD4-FITC (catalog number: 345768, clone number: SK3, BD Biosciences), CD8-V450 (catalog number: 560347, clone number: RPA-T8, BD Biosciences), CD19-PE-Cy7 (catalog number: 341113, clone number: SJ25C1, BD Biosciences), CD56-PE (catalog number: 345810, clone number: MY31, BD Biosciences), CD16-APC-H7 (catalog number: 560195, clone number: 3G8, BD Biosciences), HLA-DR-V450 (catalog number: 655874, clone number: L243, BD Biosciences) CD123-APC (catalog number: 560087, clone number: 7G3, BD Biosciences), CD196-PE (catalog number: 551773, clone number: -), CD197-PerCP-Cy5.5 (catalog number: 353220, clone number: G043H7 BioLegend San Diego, CA, United States), CD45RO-PE-Cy7 (catalog number: 560608, clone number: UCHL1, BD Biosciences), CD45RA-APC (catalog number: 550855, clone number: -, BD Biosciences), CD62L-PE (catalog number: 555544, clone number: -, BD Biosciences), CD31-PerCP-Cy5.5 (catalog number: 303132, clone number: WM59, BioLegend San Diego, CA, United States), CD127-FITC (catalog number: 560549, clone number: HL-7R-M21, BD Biosciences), CD25- APC (catalog number: 340907, clone number: 2A3, BD Biosciences).

Using the appropriate combination of the above antibodies, we distinguished the following of CD4+ or CD8+ T cell subtypes (11) **Table 1**.

**Table 1:** List of antibodies used to identify the T cell subpopulations.

among CD4+ cells	
recent thymic emigrants CD4+ cells (RTE)	CD3+ CD4+ CD45RA+ CD62L+ CD31+ CD45+
naïve CD4+ cells	CD3+ CD4+ CD45RA+ CD197+ CD45+
effector CD4+ cells	CD3+ CD4+ CD45RA+ CD197- CD45+
central memory CD4+ cells:	CD3+ CD4+ CD45RO+ CD197+ CD45+
effector memory CD4+ cells:	CD3+ CD4+ CD45RO+ CD197- CD45+
*among CD8+ cells*	
recent thymic emigrants CD8+ cells (RTE)	CD3+ CD8+ CD45RA+ CD62L+ CD31+ CD45+
naïve CD8+ cells	CD3+ CD8+ CD45RA+ CD197+ CD45+
effector CD8+ cells	CD3+ CD8+ CD45RA+ CD197- CD45+
central memory CD8+ cells:	CD3+ CD8+ CD45RO+ CD197+ CD45+
effector memory CD8+ cells:	CD3+ CD8+ CD45RO+ CD197- CD45+

**Figure 1:**
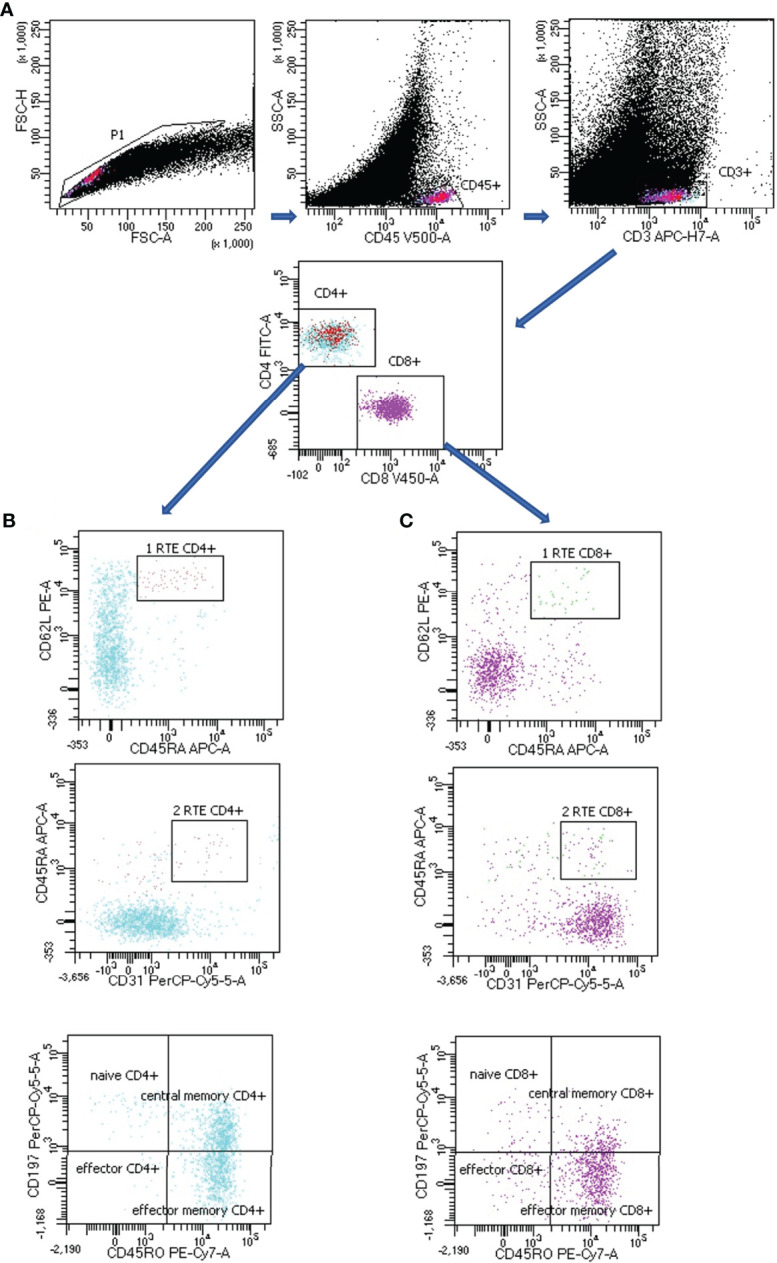
Representative T subsets gating strategy in lymph node (LN) aspirate of lung cancer patient. **(A)** CD4+ or CD8+ T lymphocytes: FSC-H vs. FSC-A plot: Gating the cells and removing clumps (greater FSC-A relative to FSC-H) and debris (very low FSC), SSC-A vs. CD45 plot: Selection of lymphocytes based on their CD45 positive properties, SSC-A vs. CD3 plot: Selection of lymphocytes T based on their CD3 positive properties. CD4 vs. CD8 plot: Selection of lymphocytes T CD4+ (turquoise) and CD8 + (violet) based on their CD4 positive or CD8 positive properties. **(B)** CD4+ T lymphocytes subsets: CD62L vs. CD45RA plot together with CD45RA vs. CD31 plot: Selection of recent thymic emigrants T CD4+ cells (RTE CD4+) based on their CD62L positive, CD45RA positive and CD31 positive properties, CD197 vs. CD45RO plot: Selection of naïve CD4+ T cells, effector CD4+ T cells, central memory CD4+ T cells and effector memory CD4+ T cells based on their CD197/CD45RO properties (the exact antigenic characterization can be found in the text in Materials and Methods section, Flow cytometry analysis). **(C)** CD8+ T lymphocytes subsets: CD62L vs. CD45RA plot together with CD45RA vs. CD31 plot: Selection of recent thymic emigrants T CD8+ cells (RTE CD8+) based on their CD62L positive, CD45RA positive and CD31 positive properties, CD197 vs. CD45RO plot: Selection of naïve CD8+ T cells, effector CD8+ T cells, central memory CD8+ T cells and effector memory CD8+ T cells based on their CD197/CD45RO properties (the exact antigenic characterization can be found in the text in Materials and Methods section, Flow cytometry analysis).

T lymphocytes subsets gating strategy in LNs of lung cancer patients is presented in **Figure 1**.

In this study the other subpopulations of T lymphocytes were also analyzed:

- Th17- related cells (CD3+ CD4+ CD45RO+ CD196+),
- T regulatory cells (Tregs CD3+ CD4+ CD25+high CD127−) and
- Tregs with expression of CD45RO+ (Tregs: CD3+ CD4+ CD25+high CD127- CD45RO+).

**Figure 2:**
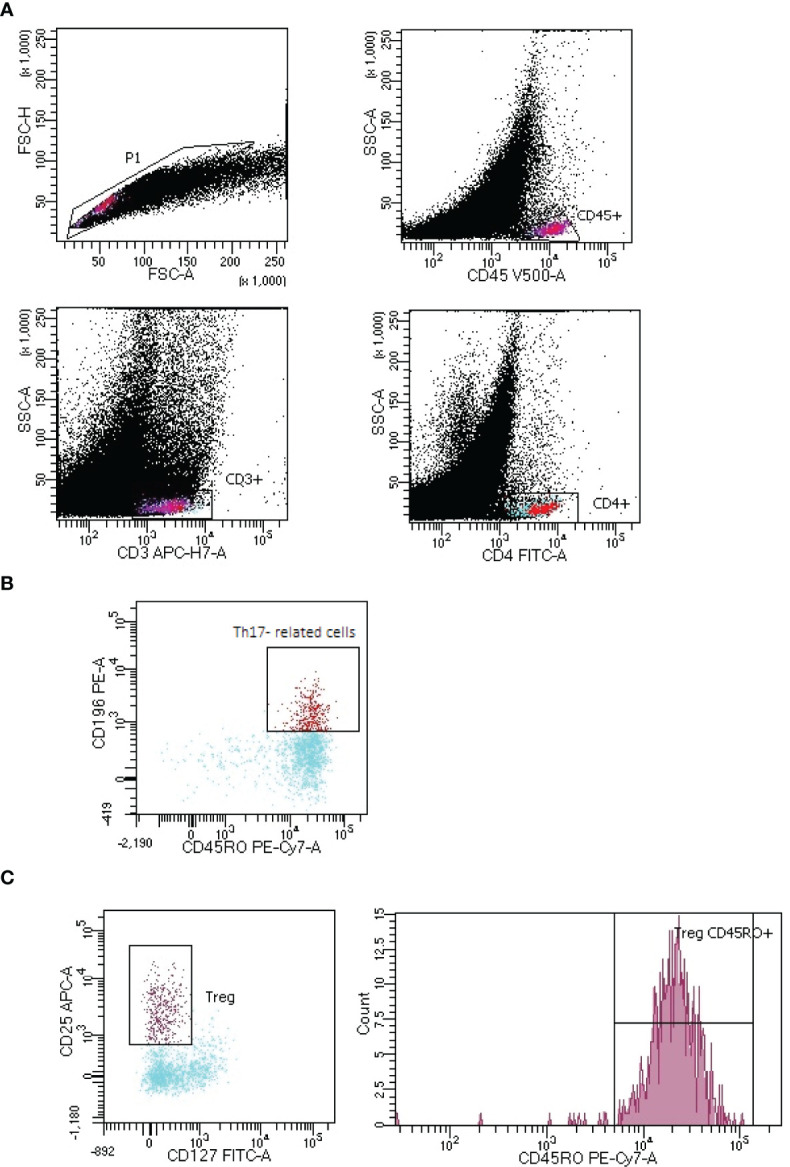
Representative gating strategy of Th17- related and T regulatory cells (Tregs) with expression of CD45RO+ in lymph node (LNs) aspirate of lung cancer patient. **(A)** CD4+ T lymphocytes:. FSC-H vs. FSC-A plot: Gating the cells and removing clumps (greater FSC-A relative to FSC-H) and debris (very low FSC), CD45 vs. SSC-A plot: Selection of lymphocytes based on their CD45 positive properties, CD3 vs. SSC-A plot: Selection of lymphocytes T based on their CD3 positive properties. CD4 vs. SSC-A plot: Selection of lymphocytes T CD4+ (turquoise) based on their CD4 positive properties. **(B)** Th17 lymphocytes: CD196 vs. CD45RO plot: Selection of Th17 cells based on their CD196 positive and CD45RO positive properties. **(C)** Tregs with CD45RO+ expression: CD25 vs. CD127 plot: Selection of Tregs based on their CD25 high positive and CD127 positive properties, count vs. CD45RO+ histogram: Selection of lymphocytes Tregs with CD45RO+ expression.

Tregs, CD45RO+ Tregs and Th17- related cells lymphocytes gating strategy in LNs of lung cancer patients is presented in **Figure 2**.

Moreover, we distinguished the following subpopulations of main leukocytes: lymphocytes (CD45+bright SSC low), lymphocytes T (CD45+bright SSC low CD3+), lymphocytes B (CD45+bright SSC low CD19+), NK cells (CD45+bright SSC low CD3- CD16+), neutrophils (CD45+ SSC bright CD16+), dendritic cells (CD45+ CD123+ bright HLA-DR+), monocytoid line cells: monocytes (in PB: CD45+bright SSC+ HLA-DR+), and macrophages (in EBUS/TBNA: CD45+bright SSC+bright HLA-DR+bright).

Internal quality control was performed daily by checking the optical detector, aligning lasers, and fluid systems using CS&T IVD Beads BD FACS Diva (BD Biosciences, San Jose, CA USA), respectively, according to the manufacturer’s guidelines.

Lymphocyte counts were obtained using a SYSMEX XN-1500 (Sysmex Corp., Kobe, Japan) haematological analyzer.

## Results

### Patients’ Characteristics

In all patients with suspicion of lung cancer, with the presence of mediastinal lymphadenopathy, a chest X-ray was performed. Computed tomography was performed showing altered LNs. EBUS/TBNA was made for cytological evaluation of the lesion and obtaining material for evaluation by flow cytometry. Histopathological examination of the changed lymph node made the final diagnosis.

There were 30 patients with newly diagnosed and confirmed lung cancer (clinical patient characteristic is presented in **Table 2**). There were 21 men (the mean age: 65 years, the range min-max: 47-79) and 9 women (the mean age: 67 years, the range min-max: 60-74). There were 13 smokers, 5 ex-smokers, and 12 never smokers in the group. Most patients in the study group were in an advanced stage of lung cancer (stage I: 5 (16.7%), II: 4 (13.3%), III: n = 12 (40.0%) and stage IV: n = 9 (30.0%)). Histological examination revealed: squamous cell lung cancer (SQCLC), 36.7%; small cell lung cancer (SCLC), 30.0%; adenocarcinoma (ADC), 23.3%; not otherwise specified (NOS), 6.7%; and adenosquamous carcinoma (ADSQ), 3.3%. Due to a small number of patients, we did not perform a comparison between the groups with different NSCLC types or between different stages of the disease.

**Table 2:** The characteristics of the investigated group.

Case	LNs metastases in cytology	Sex/age	Histological subtype	TNM	Stage	PD-L1	EGFR/ALK/KRAS	Pack years	sm/ex/non
1.	no	M/66	SQCLC	T4N1M0	IIIA	1%	x/x/x	30	sm
2.	yes	M/65	SQCLC	T1aN2M0	IIIA	0%	-/+/-	–	non
3.	yes	F/74	ADC	T4N2M2	IVB	7%	+/x/x	40	sm
4.	yes	M/62	NOS	T2N1M1	IV	1%	+/-/-	40	sm
5.	yes	M/69	ADC	T3N1M1	IV	0%	-/-/-	–	non
6.	no	M/69	ASCQ	T2aN0M0	IB	1%	-/-/-	60	sm
7.	no	F/69	SQCLC	T1N0M0	IA	x	x	–	non
8.	yes	M/64	ADC	T3N1M0	IIIA	0%	-/-/+	–	non
9.	no	F/60	NOS	T1aN1M0	IIB	x	x	–	non
10.	no	F/68	SQCLC	T3N1M0	IIIA	x	x	50	sm
11.	yes	F/67	ADC	T3N1M0	IIIA	0%	+/-/-	–	non
12.	yes	M/67	SQCLC	T4N1M0	IIIA	x	x	–	non
13.	no	M/60	ADC	T1N0M0	IA	x	x	60	sm
14.	yes	M/68	SQCLC	T3N2M1a	IV	x	x	50	sm
15.	no	M/68	SQCLC	T4N1M0	IIIA	x	x	–	non
16.	no	M/72	ADC	T2bN0M0	IIA	x	x	30	sm
17.	yes	M/47	ADC	T2aN2M0	IIIA	x	x	30	sm
18.	no	M/59	SQCLC	T3N0M0	IIB	30%	-/-/x	–	non
19.	yes	F/70	SCLC	T3N2M1	IVA	x	x	0	ex
20.	yes	M/79	SCLC	T4N2M0	IIIB	x	x	0	ex
21.	yes	M/62	SCLC	T3N1M0	IIIA	x	x	60	sm
22.	no	M/62	SQCLC	T2aN0M0	IB	x	x	30	sm
23.	no	M/64	SQCLC	T2aN0M0	IB	x	x	80	sm
24.	yes	F/69	SCLC	T3N1M1	IV	x	x	0	ex
25.	yes	M/73	SCLC	T3N1M1	IV	x	x	–	non
26.	yes	F/61	SCLC	T2N1M0	IIB	x	x	0	ex
27.	yes	M/59	SQCLC	T4N1M0	IIIA	x	x	0	ex
28.	yes	M/57	SCLC	T4N1M1	IV	x	x	–	non
29.	no	F/68	SCLC	T4N1M1c	IVB	x	x	30	sm
30.	yes	M/67	SCLC	T4N1M0	IIIA	x	x	–	non

ADC, adenocarcinoma; ADSQ, adenosquamous carcinoma; ex, ex-smoker; F, female; LN, lymph node; M, male; non, non-smoker; NOS, not otherwise specified; SCLC, small cell carcinoma; sm, smoker; SQCLC, squamous cell carcinoma;

Legend for assessing the mutation: %, activity as a percentage; +, positive; -, negative; x, without examination.

For further comparative analysis using flow cytometry, we selected patients: with the presence of metastases in LN based on histological confirmation (n= 18)and without metastases (n = 12).

There were no differences in the median proportion of main leukocyte subpopulations and T subsets between PB from patients with non-metastatic LNs and PB from patients with metastatic LNs, therefore, PB was not considered in the further analysis (**Tables S1, S2**, Supplementary data section).

### T Lymphocytes Subsets

#### CD4+ Subsets

**Figure 3:**
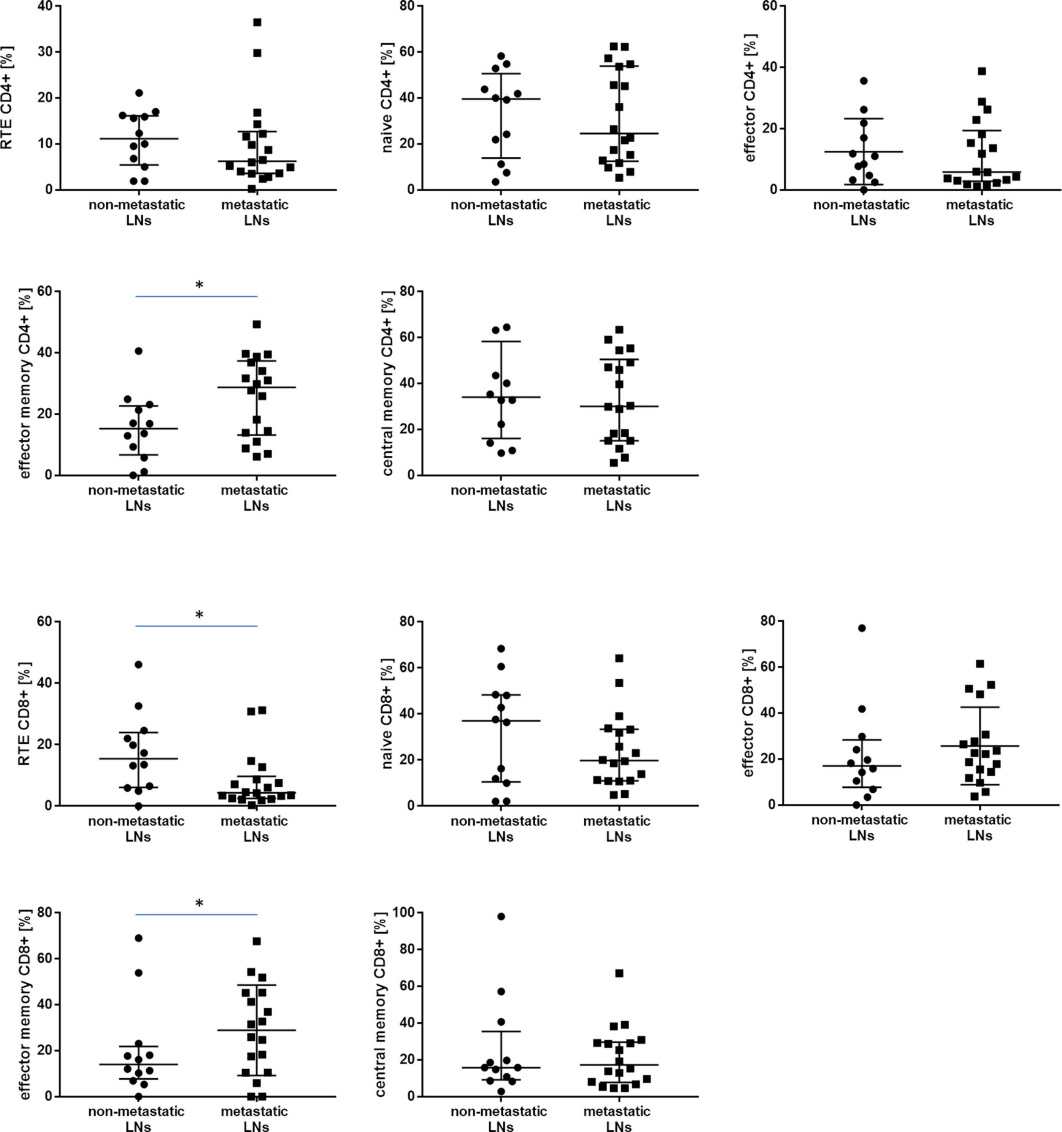
The differences in the median proportion of T lymphocytes subsets (CD4+ and CD8+): Recent thymic emigrants T cells (RTE), naïve T cells, effector T cells, central memory T cells and effector memory T cells between metastatic lymph nodes (LNs) and non-metastatic LNs of the patients with lung cancer. Graphs show the median values (Min-Max). Significant differences in the cell proportion between the non-metastatic lymph nodes (LNs) and the metastatic LNs in the Mann–Whitney U test presented as * (p<0.05).

In analyzing CD4+ T cells, we observed higher median proportion of effector memory CD4+ T cells in metastatic LNs than non-metastatic (28.7 vs. 15.3%, p= 0.047865). No other differences were observed for the CD4 subtypes of lymphocytes, such as effector, central memory, naïve, or RTE, between metastatic LNs and non-metastatic LNs (**Table 3** and **Figure 3**). The ratio of CD4 naive cells to CD4 memory effector cells did not differ between metastatic and non-metastatic LN (2.59 vs. 0.81, p> 0.05) (data not shown).

**Table 3:** Differences in the median proportion of T lymphocytes subsets in lung cancer patients between non-metastatic lymph nodes (LNs) and metastatic LNs.

[median (Q1-Q3)]	non-metastatic LNs n=12	metastatic LNs n=18	* p<0,05 The Mann–Whitney U test
cells (% of CD4+ cells)			
recent thymic emigrants (RTE) CD4+	11.1 (5.9-16.0)	6.2 (3.6-12.2)	p= 0.304764
naïve CD4+	39.6 (16.6-48.3)	24.5 (12.8-353.6)	p= 0.983383
effector CD4+	9.7 (3.9-19.4)	5.8 (3.0-18.2)	p= 0.723117
effector memory CD4+	15.3 (7.5-22.2)	28.7 (13.9-36.9)	* p= 0.047865
central memory CD4+	34.0 (18.1-53.3)	30.0 (15.1-49.2)	p= 0.661522
cells (% of CD8+ cells)			
recent thymic emigrants (RTE) CD8+	15.4 (6.2-23.3)	4.3 (2.5-8.7)	* p= 0.024561
naïve CD8+	37.0 (10.9-48.2)	19.7 (11.0-33.2)	p= 0.415000
effector CD8+	17.0 (8.6-26.9)	22.4 (14.4-30.6)	p= 0.368427
effector memory CD8+	14.0 (8.5-20.5)	28.6 (10.5-45.2)	*p= 0.045960
central memory CD8+	15.8 (9.7-30.2)	17.3 (8.0-29.2)	p= 0.851285

Data expressed as median (Q1–Q3). A *marked p< 0.05 statistically significant.

LNs, lymph nodes.

#### CD8+ Subsets

In analyzing CD8+ T cells, we observed higher median proportion of effector memory CD8+ T cells in metastatic LNs than non-metastatic LNs (28.6 vs. 14.0%, p= 0.045960). We observed lower median proportion of RTE CD8+ cells in metastatic LNs than non-metastatic LNs (4.3 vs. 15.4, p= 0.024561) (**Table 3** and **Figure 3**). The ratio of CD8 naive cells to CD8 memory effector cells did not differ between metastatic and non-metastatic LN (2.37 vs. 0.58, p> 0.05) (data not shown).

### Other T Lymphocytes Subsets

**Figure 4:**
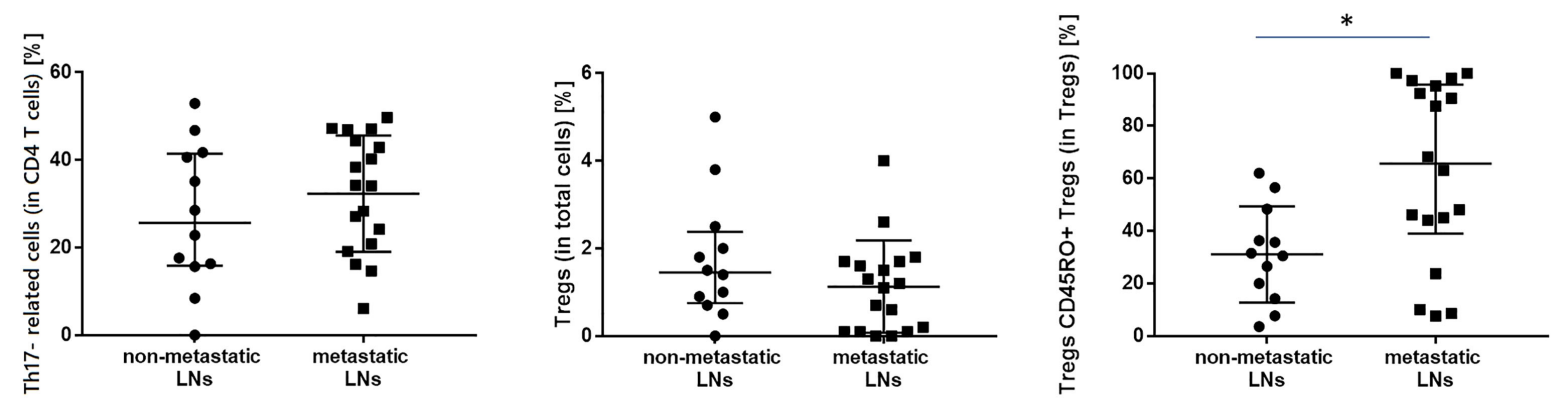
The differences in the median proportion of T regulatory cells (Tregs) (as a % of T cells), Tregs with CD45RO+ expression and Th17- related (as a % of CD4+ cells) between metastatic lymph nodes (LNs) and non-metastatic LNs of the patients with lung cancer. Graphs show the median values (Min-Max). Significant differences in the cell proportion between the non-metastatic lymph nodes (LNs) and the metastatic LNs in the Mann–Whitney U test presented as * (p<0.05).

In this study, the proportion of following T lymphocyte subsets was analyzed such as Th17- related cells, Tregs, and Tregs with expression of CD45RO+ (**Table 4**). We observed a higher median proportion of CD45RO+ Tregs in metastatic LNs than in non-metastatic LNs (65.6 vs. 31.0%, p= 0.01040, result is presented as % of CD45RO+ cells among Tregs) (**Figure 4**).

**Table 4:** Differences in the median proportion of T lymphocytes subpopulation: Th17- related and regulatory T cells with CD45RO+ expression in lung cancer patients between non-metastatic lymph nodes (LNs) and metastatic LNs.

[median (Q1-Q3)]	non-metastatic LNs n=12	metastatic LNs n=18	* p<0,05 The Mann–Whitney U test
Th17- related/T CD4+ cells	25.6 (16.0-41.1)	34.1 (20.8-44.4)	p= 0.415000
Regulatory T cells (Tregs)/total	1.4 (0.8-2.2)	1.1 (0.1-1.7)	p= 0.248642
Regulatory T cells (Tregs)/T cells	3.6 (2.2-5.7)	5.8 (3.1-8.2)	p= 0.346371
Tregs CD45RO+/among Tregs	31.0 (17.1-42.4)	65.6 (44.1-95.2)	* p= 0.015040

Data expressed as median (Q1–Q3). A *marked p< 0.05 statistically significant.

LN, lymph node.

### Correlation Between T Cells Subsets

Considering the correlations between the CD4+ T cell subsets, a significant negative correlation was observed between effector memory CD4+ cells and naïve CD4+ cells (r= -0.5, p< 0.05).

Effector memory CD8+ negatively correlated with effector CD4+ (r= -0.6, p< 0.05), with naïve CD8+ (r= -0.8, p< 0.05), and with effector CD8+ (r= -0.6, p< 0.05).

In the metastatic LN group, there was a significantly negative correlation between the proportion of effector CD4+ cells and Tregs with CD45RO+ expression (r = -0.6, p < 0.05).

In the metastatic LN group, there was a high positive correlation between the proportion of effector memory CD8+ and Tregs with CD45RO+ expression (R = 0.6, p < 0.05).

There was no correlation between T cell populations and Th17- related cells.

**Figure 5:**
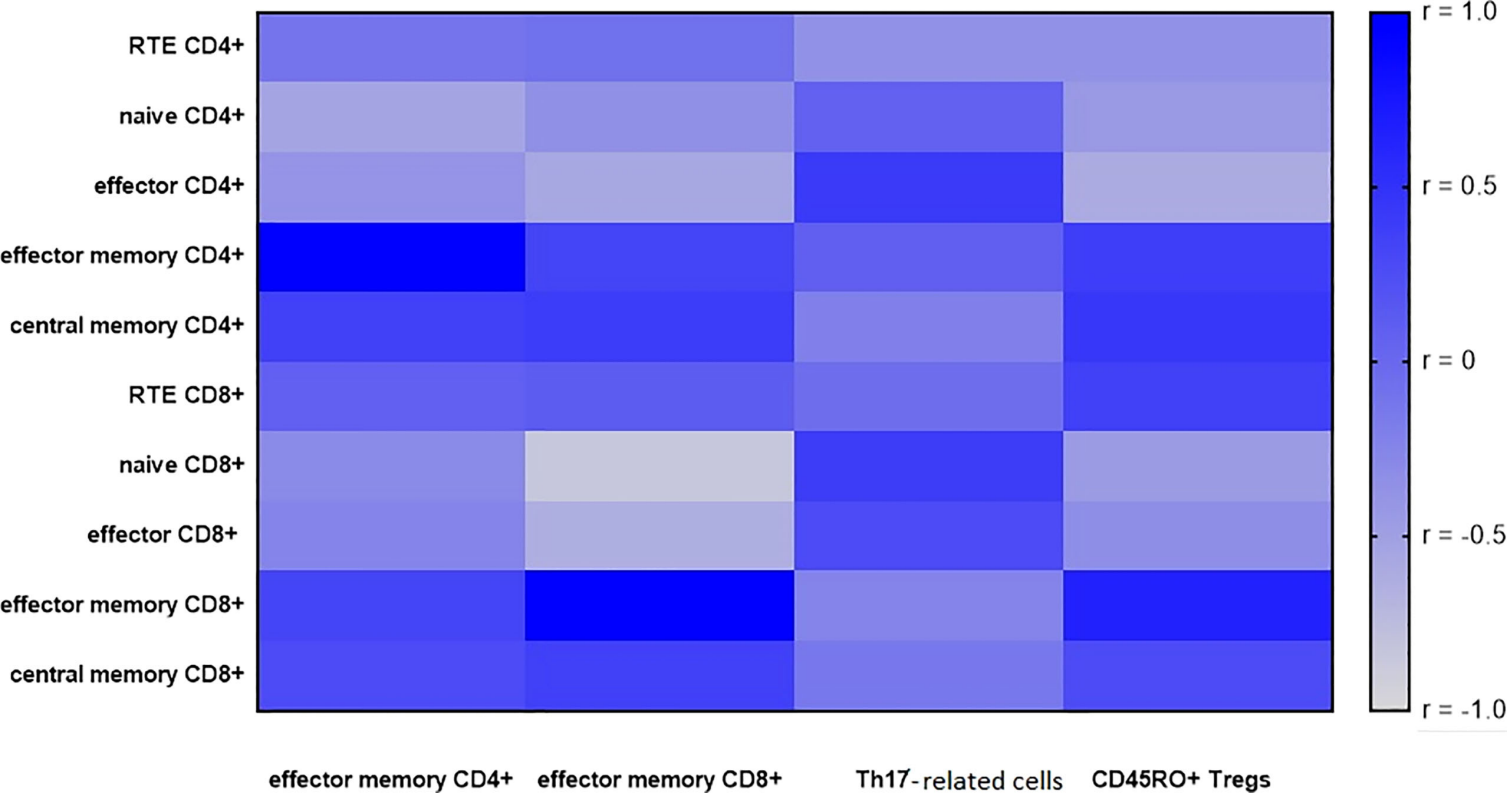
A heatmap of Spearman correlation coefficients for the effector memory T cells (CD4+ and CD8+), Th17- related and Tregs with CD45RO+ expression with T subsets in metastatic lymph nodes (LNs). Correlations with an absolute value more than 0.5 are associated with p < 0.05, blue—positive correlations, gray—negative correlations.

All correlations between the proportion of effector memory CD4+, effector memory CD8+ cells, Th17- related cells, and Tregs with CD45RO+ expression with T subset in metastatic LNs are presented in heat maps (**Figure 5**).

### Leukocyte Subsets in Lymph Nodes

We also analyzed the main leukocyte profile in LNs in patients with lung cancer depending on presence of metastases in LNs. We observed a lower median proportion of lymphocytes in metastatic LNs than in non-metastatic LNs (**Table 5**). There was a higher median proportion of monocyte/macrophage lineage cells and dendritic cells (DCs) in metastatic LNs than non-metastatic LNs.

**Table 5:** The differences in the median proportion of leukocytes and main lymphocytes subpopulation between non-metastatic lymph nodes (LNs) and metastatic LNs.

% of all leukocytes	non-metastatic LNs n=12	metastatic LNs n=18	p < 0.05 * Mann-Whitney U test
Lymphocytes	72.2 (47.0-90.9)	51.9 (29.3-60.1)	* p= 0.034627
Lymphocytes T	50.0 (34.2-61.9)	28.4 (18.7-51.8)	p= 0.071600
CD4 cells	31.2 (14.9-49.9)	16.1 (9.8-24.8)	p= 0.064938
CD8 cells	13.9 (8.2-19.3)	9.9 (6.6-12.7)	p= 0.368427
Ratio CD4/CD8	2.4 (1.1-5.0)	1.8 (1.3-2.1)	p= 0.231598
NKT cells	0.2 (0.0-1.0)	0.8 (0.1-2.0)	p= 0.123589
Lymphocytes B	16.8 (3.9-24.9)	5.9 (2.4-13.1)	p= 0.248642
NK cells	1.8 (1.2-3.3)	3.3 (1.4-7.8)	p= 0.346371
Neutrophils	25.7 (7.1-45.3)	39.2 (28.4-62.4)	p= 0.134493
DCs	0.2 (0.0-0.3)	0.7 (0.2-1.6)	* p= 0.005782
MMLCs	1.4 (0.8-3.6)	5.7 (2.8-8.4)	* p= 0.004291

Data expressed as median (Q1–Q3). A *marked p< 0.05 statistically significant.

LN, lymph node; MNLCs, Monocytoid line: monocyte/macrophage lineage cells.

Cells not expressing CD45 antigen (non-hematopoietic cells), including tumor cells, were not included in this study.

## Discussion

In this study, we focused on assessing the LNs cell profile of lung cancer patients with an emphasis on distinguishing the dominant status of T-cell subsets. We focused on the possibility of a practical and accessible assessment of the main subpopulations of T lymphocytes, before possible immunotherapy, as a potential biomarker. Despite the fact that there are many mechanisms which allow cancer cell to escape from immune surveillance (45, 46), the cellular defense is the basis of anti-cancer resistance, used among others, in immunotherapy. It is known that lymphocytes T are the most essential inflammatory cells infiltrating the tumor, exerting a direct cytotoxic effect, or leading to tumor lysis by cytokine release (47–49).

We assumed that it would be important to analyze the process of maturation and differentiation of T cells in LNs of lung cancer patients. The results of our study show, for the first time, the important differences between metastatic and non-metastatic LNs evaluated using EBUS/TBNA and flow cytometry.

### T Lymphocyte Subsets Profile in Lung Cancer LNs

In our study the T lymphocytes subsets profile revealed higher median proportion of effector memory CD4+ T cells and effector memory CD8+ T cells in metastatic LNs than non-metastatic LNs.

Moreover, effector memory T cells turned out the dominant population among the total CD8+ cells population. Additionally, we found lower median proportion of RTE CD8+ cells in metastatic than non-metastatic LNs.

It is known that effector memory cells produce many inflammatory cytokines and their most important role is related to the activation of the immune response taking place in peripheral lymphoid organs. These cells do not pass through the lymph nodes but flow to the inflamed sites (12). The function of effector memory T cells depends on the presence of a persistent antigen in the organism and when reaching the site of inflammation, they act through the secretion of cytokines or by a cytotoxic effect (50). Effector memory T cells express higher levels of receptors responsible for migration to inflamed tissues and have a stronger immediate effector function than central memory T (12, 51). In several solid tumors, tumor-specific T cell memory responses likely play an important role in keeping tumors in check, limiting cancer cell dissemination and reducing risk of relapse.

Our results may indicate the cytotoxic reaction to cancer cells in metastatic LNs. Other researchers confirm the presence of effector lymphocytes at the site of the ongoing neoplastic process and their prognostic importance. Our present and former findings indicate specificity of the character of the environment of the metastatic tumor site (52–55). The high number of CD8+ CD45RO+ effector memory T cells found in the primary tumor lesion significantly correlates with a positive clinical outcome in various cancers. In a meta-analysis of 25 published studies with 4,720 patients, researchers assessed the prognostic effect of tumor infiltrating CD45RO+ T cells in human solid tumors (56). It was shown that the infiltration of CD45RO+ T cells was significantly associated with improved overall survival (OS) and disease-free survival (DFS) in all types of solid tumors. Moreover, the high density of CD45RO+ T cells inside the tumor was inversely correlated with the TNM stage of the solid tumor.

Recent research studies on cancer immunotherapy have focused on the use of anti-tumor CD8+ cytotoxic T-cell responses. Principe N. et al. suggest that increased effector memory tumor antigen-specific cytotoxic lymphocytes T CD8+, in the presence of reduced immunosuppression within tumors, is part of a successful immune check point therapy response (57). Other researchers propose an assessment the T central memory to T effector cells (Tcm/Teff) ratio as a predictive biomarker of response to checkpoint inhibitors in NSCLC patients (58).

Interestingly, in our study, despite the expected increase in effector memory cells, we also noticed a decrease in the number of naive RTE CD8+ cells in metastatic LNs. This phenomenon may mean that the tumor has silenced the immune response, stopped the influx of cells into the LNs, or redirected the body’s reaction to the effector ones.

### Other T Lymphocyte Subsets

There is evidence that tumor cells form an immunosuppressant TME, including factors secreted by tumor or stromal cells and inhibitory immune cells such as myeloid suppressor cells (MDSC) and Tregs, affecting the activity of T cells (59). The development of the tumor and the spread of tumor cells is facilitated by the so-called immune tolerance, i.e., the lack of response to antigens. Tregs play the most important role in immune surveillance and tolerance mechanisms. The number of circulating Tregs are increased in patients with cancer, including lung cancer (25, 26, 60). Interestingly, in the present study, the proportion of Treg cells did not differ between metastatic and non-metastatic LNs, but the expression of the CD45RO+ antigen on these cells differed significantly. The proportion of CD45RO+ Tregs was higher in the metastatic than in the non-metastatic LNs. This observation confirms the importance of studies of various Tregs subtypes, showing different proliferation and migration potential. Amarillo D. et al. has shown that there was a significant difference between the percentage of memory Tregs (CD45RO+) in control patients and lung cancer patients (19.52 vs. 86.77% p <0.001) and confirmed that circulating Tregs are a potential prognostic factor in lung cancer patients (61).

It was found that increased tumor infiltrating Tregs are associated with poor overall survival in SCLC (62). Moreover, Kotsakis A. et al. determined that the increased presence of terminal effector Tregs predicts improved overall and progression-free survival, whereas an increase in naïve or effector Tregs number was associated with worse survival (25). The expression of the CD45RO marker on effector Treg (CD4+ CD25high CD127low CD45RO+) suggests activation and ability to proliferate but also could indicate a short-lived, terminally diverse population that is rapidly fragmenting and declining (63, 64). As confirmed by the above observation, the collective study of the Tregs population in metastatic LNs may not be sufficient, without precise differentiation, and the role of these cells seems to be very complex in lung cancer patients.

In this study, no difference in the median proportion of Th17- related cells was observed between the metastatic and non-metastatic LNs. Th17 participate in antimicrobial and fungal immunity, allergic, and autoimmune diseases, as well as anti-inflammatory responses (65, 66). Th17 cells are key producers of IL-17, and this cytokine is known to contribute to the induction of lung cancer prometastatic factor expression (67). Many studies reveal the potential functional effects of IL-17 on cancer progression and metastasis. In humans, the increased density of IL-17-positive cells in NSCLC tumors correlated with the density of lymphatic vessels. In addition, loss of IL-17 has been shown to reduce metastasis (68, 69).

The role of Th17 cells in promoting or inhibiting various human cancers seems to be context-dependent (70, 71). The influence of environmental cues may be one of the major determinants to modulate Th17 cell recruitment and function. Recently, growing evidence indicates that tumor-intrinsic genetics determine the corresponding immune profile and Th17 cell function.

Numerous studies have shown that the cytokine IL-17, directly or indirectly, promotes tumor angiogenesis and tumor cell proliferation and inhibits apoptosis by activating inflammatory signaling pathways. Thus, IL-17 contributes to the progression of lung cancer (69, 72, 73).

The above-mentioned studies confirm the robust role of Th17 cells in the course of lung cancer. In our study we found no differences in the proportion of Th17- related cells in non-metastatic and metastatic LNs. Perhaps not number of these cells alone is important, but their degree of stimulation and the ability to produce IL-17. The lack of differences in our work between metastatic and non-metastatic LN may also result from the imprecise phenotypic definition of these cells and the lack of application of functional methods, which is a limitation of our study.

In our study, the main leukocyte population was also divided for the purpose of precisely defining the T lymphocyte subpopulations. However, it was not the subject of the present study but it should be emphasized that the differences in the number of DCs and monocyte/macrophage lineage cells between the metastatic and non-metastatic LNs were presented. We showed higher median proportion of monocyte/macrophage lineage cells, and DCs in metastatic LNs than non-metastatic LNs. This observation confirms the current state of knowledge that, in addition to lymphocytes, important cells within the tumor area are antigen presenting cells (APC), such as DCs and monocytes. In our other work, we present the subtypes of DCs cells (55) and discuss their involvement in metastatic processes together with heterogeneous monocytes (74).

### Relationships Between T Lymphocytes Subset in Metastatic LNs

Next, we analyzed the relationship between the effector memory CD4+ and CD8+ cells with other subsets of T cells, CD45RO+ Tregs and Th17- related using correlation in metastatic LNs. Presented correlations confirm the important role of effector memory cells, mainly effector memory CD8+ cells in lung cancer. We noticed more correlations among the subpopulations of T lymphocytes with CD8+ cells than with CD4+ cells in LNs with metastases of lung cancer patients. We showed negative correlation of effector memory T CD8+ cells with effector CD4+, with naïve CD8+, with effector CD8+ cell.

By analyzing the differences between metastatic and non-metastatic LNs, we found that effector memory T cells are an interesting parameter to use for the evaluation of immune status patients with lung cancer. Given the relationships in metastatic LNs between T cell subpopulations as assessed by the correlation, we can also hypothesize about the cytotoxic response of the organism to the tumor and the importance of effector memory cells. Interestingly, in our study, we also observed a high positive correlation between effector memory CD8+ cells and CD45RO+ Tregs. Perhaps these two parameters together differentiate patients with and without metastases and a positive correlation with each other could be used in the evaluation of patients with lung cancers.

To summarize, the LN is known to be a common site of metastasis and lymph node disease predicts increased mortality in many types of cancer. Moreover, LNs are critical in initiating anti-cancer immune responses (75, 76). Metastatic cells in lymphoid organs are important in the induction of anti-cancer CD8+ T cells and tumor rejection (77, 78). Understanding the mechanisms of survival, tumor growth, and immune system response in LNs is key to designing an effective therapy to eradicate LN metastasis. It has been hypothesized that the correlation of effector memory cells with other subsets of T lymphocytes would allow recognition of significant cells and their relationship in the course of lung cancer all of which fits in the progress of prognosis assessment and the determination of new immunological therapeutic points.

Based on the above results, we confirm that using EBUS/TBNA methods with flow cytometry is possible to obtain diagnostic material from the LN, which in the metastatic LNs, have a characteristic T-lymphocyte profile.

## Conclusions

In conclusion, our findings show that assessment T cell profile in relation to other T cell subsets by flow cytometry analysis of EBUS/TBNA samples can provide insight into the immune status of metastatic LNs and thus reflect local TME. It is possible during diagnostic procedure to inform about the state of the anti-tumor immune response and further identify potential biomarkers of clinical response to modern therapies of lung cancer.

## Data Availability Statement

The raw data supporting the conclusions of this article will be made available by the authors, without undue reservation.

## Ethics Statement

The studies involving human participants were reviewed and approved by Military Institute of Medicine Ethics Committee: 25/WIM/2018. The patients/participants provided their written informed consent to participate in this study.

## Author Contributions

IK and ER: Conceptualization and methodology; IK, RS, and JB: Data curation; IK and ER: Writing—original draft preparation; IK, KJ-R, PR, and JD-K: Writing—review and editing; AR: Visualization; JD-K: Supervision. All authors contributed to the article and approved the submitted version.

## Funding

This study was funded by Military Institute of Medicine, grant number 559.

## Conflict of Interest

The authors declare that the research was conducted in the absence of any commercial or financial relationships that could be construed as a potential conflict of interest.

## Publisher’s Note

All claims expressed in this article are solely those of the authors and do not necessarily represent those of their affiliated organizations, or those of the publisher, the editors and the reviewers. Any product that may be evaluated in this article, or claim that may be made by its manufacturer, is not guaranteed or endorsed by the publisher.
