# Supplementary material for: Effector Memory T Cells and CD45RO+ Regulatory T Cells in Metastatic vs. Non-Metastatic Lymph Nodes in Lung Cancer Patients
Source: Front Immunol. 2022 May 2;13:864497. doi: 10.3389/fimmu.2022.864497 (PMC9108231; doi:10.3389/fimmu.2022.864497)
Supplement: Supplementary file 1 [file Table_1.docx]

Supplementary Material

# Supplementary Tables

**Table S1.** Differences in the median proportion and median absolute count of leukocytes and main lymphocytes subpopulation between peripheral blood (PB) from patients with non-metastatic lymph nodes (LNs) and PB from patients with metastatic LNs. Data expressed as median (Q1–Q3). A * marked p< 0.05 statistically significant.

| % of all leukocytes | **PB non-metastatic**  **LNs**  **n= 12** | **PB metastatic**  **LNs**  **n=18** | **p < 0.05 ***  **Mann-Whitney U test** |
| --- | --- | --- | --- |
| Lymphocytes | 36.7 (21.1-43.1) | 23.3 (15.0-33.9) | p= 0.083554 |
| Lymphocytes T | 25.8 (15.3-31.7) | 17.2 (11.7-24.0) | p= 0.133831 |
| CD4 cells | 17.2 (9.0-19.8) | 10.0 (4.2-13.1) | p= 0.083554 |
| CD8 cells | 7.5 (6.6-13.0) | 6.8 (4.6-10.5) | p= 0.386939 |
| Ratio CD4/CD8 | 1.2 (1.2-2.4) | 1.3 (0.9-1.9) | p= 0.550075 |
| NKT cells | 0.1 (0.0-0.4) | 0.1 (0.0-0.3) | p= 0.947159 |
| Lymphocytes B | 2.9 (2.1-7.5) | 1.8 (1.1-4.0) | p= 0.220433 |
| NK cells | 3.9 (2.9-8.3) | 2.3 (1.0-5.6) | p= 0.075554 |
| Neutrophils | 54.0 (49.3-73.0) | 71.3 (53.6-78.2) | p= 0.159282 |
| DCs | 0.088 (0.013-0.115) | 0.041 (0.024-0.115) | p= 0.842373 |
| Monocytes | 6.7 (6.0-9.2) | 7.1 (4.9-8.5) | p= 0.947159 |
| k/µl |  |  |  |
| Lymphocytes | 3108 (1742-3570) | 1857 (1129-2936) | p= 0.237969 |
| Lymphocytes T | 2221 (1344-2495) | 1222 (856-2200) | p= 0.237969 |
| CD4 cells | 1272 (716-1437) | 706 (519-1275) | p= 0.173257 |
| CD8 cells | 699 (373-1149) | 525 (356-956) | p= 0.362845 |
| Ratio CD4/CD8 | 1.2 (1.2-2.4) | 1.3 (0.9-1.7) | p= 0.550075 |
| NKT cells | 9.8 (0.0-48.0) | 9.0 (0.0-21.8) | p= 0.842373 |
| Lymphocytes B | 271 (134-397) | 146 (102-341) | p= 0.386939 |
| NK cells | 431 (215-768) | 224 (109-598) | p= 0.083554 |
| Neutrophils | 4665 (3040-6962) | 5586 (4642-7047) | p= 0.159282 |
| DCs | 5 (1-12) | 4 (2-14) | p= 0.982376 |
| Monocytes | 556 (391-945) | 638 (429-874) | p= 0.877092 |

Abbreviation: LN, lymph node;

**Table S2.** Differences in the median proportion of T lymphocytes subpopulation of T cells in lung cancer patients between peripheral blood (PB) from patients with non-metastatic lymph nodes (LNs) and PB from patients with metastatic LNs. Data expressed as median (Q1–Q3). A * marked p< 0.05 statistically significant.

| [median (Q1-Q3)] | **PB non-metastatic LNs**  **n=12** | **PB metastatic**  **LNs**  **n=18** | * p<0,05  The Mann–Whitney U test |
| --- | --- | --- | --- |
| cells (% of CD4+ cells) | | | |
| recent thymic emigrants (RTE) CD4+ | 9.6 (5.9-16.2) | 10.3 (8.8-20.7) | p= 0.555432 |
| naïve CD4+ | 29.4 (22.1-46.4) | 33.8 (27.1-40.6) | p= 0.688786 |
| effector CD4+ | 3.9 (1.5-6.1) | 2.4 (1.3-7.1) | p= 0.795635 |
| effector memory CD4+ | 13.9 (5.6-21.1) | 16.4 (12.4-24.3) | p= 0.464182 |
| central memory CD4+ | 47.8 (32.8-51.7) | 41.5 (35.6-48.9) | p= 0.435657 |
| cells (% of CD8+ cells) | | | |
| recent thymic emigrants (RTE) CD8+ | 11.5 (7.6-18.0) | 7.6 (6.0-17.7) | p= 0.435657 |
| naïve CD8+ | 17.6 (12.9-28.2) | 11.1 (7.9-18.4) | p= 0.132742 |
| effector CD8+ | 56.1 (28.3-62.4) | 50.1 (33.0-56.7) | p= 0.654352 |
| effector memory CD8+ | 13.4 (9.3-22.1) | 21.2 (13.1-34.8) | p= 0.064177 |
| central memory CD8+ | 12.9 (7.5-14.5) | 13.5 (9.6-18.5) | p= 0.464182 |
| Subpopulation of T cells / % of different cells | | | |
| Th17- related cells/ T CD4+ cells | 33.5 (24.0-50.7) | 27.4 (21.5-36.7) | p= 0.286222 |
| Regulatory T cells (Tregs)/ total | 0.450 (0.200-0.900) | 0.300 (0.200-0.600) | p= 0.620606 |
| Regulatory T cells (Tregs)/ T cells | 3.3 (2.0-4.1) | 3.7 (2.5-5.3) | p= 0.464182 |
| Tregs CD45RO+/Tregs cells | 86.1 (68.8-88.6) | 85.2 (79.9-95.1) | p= 0.356195 |

Abbreviation: LN, lymph node;
